# Hepatitis E Vaccines Updates

**DOI:** 10.3390/vaccines12070722

**Published:** 2024-06-28

**Authors:** Christopher Hartley, Paul Wasuwanich, Trung Van, Wikrom Karnsakul

**Affiliations:** 1 The Department of Pharmacy, The Johns Hopkins Hospital, Baltimore, MD 21287, USA; 2 University of Florida College of Medicine, Gainesville, FL 32610, USA; 3 Department of Pharmacology and Physiology, Morsani College of Medicine, University of South Florida, Tampa, FL 33612, USA; tvan6@usf.edu; 4 Pediatric Liver Center, The Department of Pediatrics, The Johns Hopkins Hospital, Baltimore, MD 21287, USA; wkarnsa1@jhmi.edu

**Keywords:** hepatitis E, emerging vaccines, clinical trial review

## Abstract

The development of a hepatitis E vaccine is imperative given its prevalence and the heightened risk it poses to specific populations. Hepatitis E virus infection, though often self-limiting, poses a significant threat to pregnant individuals and immunocompromised populations. This review delves into the historical trajectory of hepatitis E vaccine development and explores its potential impact on at-risk populations. Historically, efforts to formulate an effective vaccine against hepatitis E have been underway to mitigate the severity of the disease, particularly in regions where the infection is commonplace. As a self-limiting disease, the necessity of a vaccine becomes more pronounced when considering vulnerable demographics. Pregnant individuals face heightened complications, with potential adverse outcomes for both mother and child. Similarly, immunocompromised individuals experience prolonged and severe manifestations of the infection, necessitating targeted preventive measures. This review aims to provide a comprehensive overview of the milestones in hepatitis E vaccine development. By examining the historical progression, we aim to underscore the critical need for a vaccine to safeguard not only the general population but also those at elevated risk. The elucidation of the vaccine’s journey will contribute valuable insights into its potential benefits, aiding in the formulation of informed public health strategies to combat hepatitis E effectively.

## 1. Introduction

Hepatitis E, stemming from the hepatitis E virus (HEV), is a widespread liver ailment globally, contributing to around 20 million cases and over 44,000 deaths annually [1]. Nevertheless, its impact is notably more severe in developing nations, such as India and Bangladesh, compared to developed nations. HEV comprises five genotypes relevant to human illness: genotypes 1, 2, 3, 4, and 7. Among these, genotype 1 is more severe clinically and prevalent in developing regions and is primarily transmitted through the fecal–oral route. Genotype 2 is similar to genotype 1 in clinical presentation and transmission but is less common. Conversely, genotype 3 typically manifests as asymptomatic to mild and is primarily transmitted zoonotically from pigs and other animals [2,3]. Genotype 4 is similar to genotype 3 but is limited to a few areas globally, most notably China. Current zoonotic reservoirs include deer, pig, and wild boar [4]. Replication occurs primarily in the small intestines, lymph nodes, colon, and liver [5].

HEV infection may lead to conventional hepatic complications like jaundice, abdominal pain, and fulminant hepatitis, alongside associated extrahepatic complications such as neuralgic amyotrophy, Guillain–Barre syndrome, glomerulonephritis, acute pancreatitis, thrombocytopenia, and cryoglobulinemia [6].

In pregnant women, particularly in developing nations, HEV infection involving genotype 1 often leads to adverse maternal and fetal outcomes. A systematic review by Bergløv et al. revealed median mortality rates for HEV infection during pregnancy of 26% (IQR: 17–41%) for the mother, 33% (IQR: 19–37%) for the fetus, and 8% (IQR 3–20%) for the neonate [7]. Bergløv et al. also noted a common occurrence of fulminant hepatic failure, ranging from 9.1% to 70% (median 45.3%), and prevalence of preterm labor ranging from 2.22% to 90.4% (median 51.9%) [7]. Neonatal outcomes in pregnancies complicated by HEV infection are seldom documented. Low birth weight was a common complication observed in 57.4% to 86.7% of infants born to mothers with HEV infection [8,9,10]. Vertical transmission rates of HEV ranged from 27.9% to 78.9% [7].

In the United States, hepatitis E is relatively underexplored, and vaccine development for the disease has not been a priority. Historically, nearly all HEV infections in the United States have been associated with the milder HEV genotype 3 [11,12,13,14]. A recent study by Wasuwanich et al. focused on hepatitis E-related hospitalizations in the United States between 2010 and 2017, noting an increase in hospitalization rates over that period [15].

For over a decade, Hecolin (HEV 239), a highly effective vaccine produced by Xiamen Innovax Biotech Co., Ltd. (Xiamen, China), has been available in China (since 2011) and more recently in Pakistan. However, the company has not yet sought World Health Organization (WHO) pre-qualification review for the vaccine. In May 2015, the WHO released a hepatitis E vaccine position paper based on advice from its Scientific Advisory Group of Experts (SAGE) on immunization [16]. Acknowledging the significant public health threat posed by hepatitis E, especially among vulnerable populations including pregnant women and individuals in displaced persons’ camps, SAGE regarded HEV 239 as a promising vaccine. However, it highlighted substantial data gaps regarding the global incidence of HEV infection and disease, preventing a recommendation for routine use. Instead, it suggested that national authorities assess and decide on vaccine deployment based on local epidemiology, especially in high-risk scenarios such as outbreaks. The WHO proposed specific areas of further investigation for HEV 239, including its use in pregnant women, individuals under 16 years, the elderly, immunocompromised individuals, and those with chronic liver disease. Additionally, the paper recommended evaluating vaccine impact during outbreaks and exploring alternative dosing schedules suitable for reactive use during outbreaks and concurrent administration with other vaccines. Finally, despite HEV having only one serotype, the WHO noted that vaccine efficacy in the Phase 3 trial had been demonstrated solely against genotype 4 [16].

After the release of the 2015 WHO position paper, numerous clinical trials have been undertaken to broaden the clinical understanding of HEV 239 across diverse populations and to fill in certain knowledge gaps. Concurrently, significant HEV outbreaks have transpired since 2015, leading to avoidable illness and death [17,18]. However, in a notable development, the vaccine was utilized as a public health measure in an outbreak scenario outside of China for the first time in March 2022, in South Sudan [19].

The 2015 WHO position paper acknowledged special circumstances where the HEV vaccine could be beneficial, such as during outbreaks, but left decisions on its use to local authorities [16]. Despite attempts to deploy HEV 239 in outbreaks, these efforts were unsuccessful, highlighting the need to understand country-level barriers to its use. One common challenge is delayed identification of HEV due to limited diagnostics. There are often delays in decision-making regarding vaccine use even after outbreak declaration, partly due to countries’ independent evaluation processes. Additionally, technical gaps in some countries hinder quick decision-making, which pre-qualification could alleviate. Low awareness of HEV 239 among regulators, policymakers, and affected communities further complicates timely risk–benefit assessments. To address these barriers, proactive preparedness sessions on HEV and HEV 239 should be conducted with public health and regulatory leaders in high-risk regions [20].

## 2. Materials and Methods

We conducted a systematic search on PubMed and Google Scholar for research articles with the main keywords “hepatitis E” and “vaccine”. Non-English research articles without English translations were not reviewed. All relevant research articles found in the search were reviewed. Recent publications were favored; however, publication dates were not used as an exclusion factor [20].

## 3. Current Vaccines

There are two major vaccines currently under research: Hecolin (HEV 239), which is sponsored by Xiamen University, and SaR 56 kDa, sponsored by the United States army in collaboration with the pharmaceutical company GlaxoSmithKline (GSK). HEV 239 is a recombinant vaccine that was tested in healthy men and women aged 16 to 65 between 2007 and 2009 as a double-blind, randomized control trial in China [21]. There were 112,604 persons who were randomized to receive either the HEV vaccine (30 micrograms of purified antigen in 0.8 mg aluminum hydroxide in 0.5 mL buffered saline) or a placebo (already approved hepatitis B vaccine in 0.5 mL aluminum hydroxide). This was a 3-dose vaccine series at 0, 1, and 6 months. The primary endpoint was prevention of hepatitis E in participants who received three doses of vaccine 12 months after receiving their final vaccine dose. There were 97,356 persons who received all three vaccines, allowing them to be included in the primary endpoint. After two doses, 14 patients developed HEV in the placebo group, and after the third dose, there were 15 participants that developed HEV. The group that received HEV 239 had an efficacy of 100% in prevention of HEV. The adverse effects overall were mild and were similar between both groups, and adverse effects that were severe were reviewed by the data and safety monitoring board, which concluded that they were not due to the vaccine [21]. Hecolin is a recombinant, non-live vaccine and therefore mainly produces an antibody-mediated response rather than a cellular-mediated response [22].

A letter to the editor from Wu and colleagues from the clinical trial paper further defined safety in pregnant patients [23]. There were 37 pregnant patients in the HEV239 group (receiving a total of 52 doses) and 31 patients in the placebo group (receiving a total of 46 doses). There were no serious adverse effects noted in either group, no spontaneous abortions occurred, and no babies were born with congenital anomalies. Weights, lengths, and gestational age differences were neither clinically nor statistically significant. None of the patients developed hepatitis E [23]. More recently, a cost-effectiveness paper was written for women of child-bearing age in China by Xia and colleagues [24]. Their paper showed a decreased disability-adjusted life-year (DALY), while screening patients using their Wantai pharmaceutical anti-HEV rapid test produced the greatest cost effectiveness. They would then vaccinate patients who were anti-HEV negative. Vaccinating using their decision tree-Markov model versus vaccinating all individuals had a difference in cost of less than one for the gross domestic product (GDP) versus three times the GDP [24].

## 4. Vaccine Development

The vaccine candidates are assumed to work against all genotypes of HEV that infect humans (genotype 1–4), but the vaccines are not tested against all four genotypes. While we are highly confident that the vaccines will work similarly across the HEV genotypes, as there is only one serotype, there is no hard evidence for this in clinical trials. Hecolin, for instance, was developed from an antigen from HEV genotype 1 and has been tested with success in clinical trials in areas endemic for HEV genotype 4. Hecolin was tested in monkeys that were infected with HEV genotype 1 and was found to be successful [25]. There are ongoing studies of Hecolin in Bangladesh, where genotype 1 is endemic [26,27]. There are no studies of Hecolin on genotype 2 or 3 at this time.

Despite the discovery of HEV nearly 40 years ago, there are still many questions related to how HEV mechanistically enters susceptible cells. Researchers have learned through years of research that lipids on the cell help with transmission and that ORF2 and ORF3 phosphoproteins on the virus are required for transmission [5,28,29]. Despite this, when lipid solvents as well as detergents with anti-phosphoprotein properties are utilized to try to decrease HEV progenies, there is minimal change in the infectivity to HEV, indicating that phosphoproteins and lipids on the cells are not the only transmission technique [5,29]. Mutation rate estimations are challenging to determine due to the transient infection that HEV causes, as well as its ability to spread between both human and animal hosts [5].

The HEV239 vaccine was first tested in rhesus monkeys by showing a bacterial peptide E2, which was placed on the structure of the HEV protein, protected against hepatitis E virus genotype 1 [29,30]. When this was tested in mice, it was not effective as a vaccine, but led to HEV239 through the addition of a 26 amino acid (AA) extension at the E2 site of the HEV. HEV 239 showed a similar immune response to the original E2 peptide vaccine, but with the addition of the 26 AA, it was able to prolong antibody response [30]. The 56 kDa vaccine was developed by inoculating HEV into insect cells, which then undergo proteolytic post-translational modification to create the 56 kDa vaccine [31,32,33].

## 5. Current Studies

Upon reviewing Clinicaltrials.gov, there are currently 15 studies using the keyword “hepatitis E vaccine”, which are summarized in Table 1. 

### 5.1. Completed

#### Completed Studies with Results

An open, pared trial of recombinant hepatitis E vaccine (Escherichia Coli) Hecolin^®^ in chronic hepatitis B patients on clinical stability (NCT02964910) was sponsored by Xiamen Innovax Biotech Co. in Weihai, Shandong, China, and had 475 participants [42]. This Phase 4 clinical trial was to evaluate efficacy in patients receiving three doses at 0–1–6 months [42]. In both groups, nearly all participants who received the three vaccines (n = 188 chronic Hep B patients, n = 196 healthy volunteers) seroconverted 1 month after the last vaccine (97.9% and 100%) [42]. There were similar percentages clinically and no statistically significant differences in adverse effects between the two groups (39.1% and 40.4%, *p* = 0.778) [42].

A Phase 1, double-blinded, placebo-controlled clinical trial to evaluate the safety, reactogenicity, and immunogenicity of HEV-239 (Hecolin^®^) in a healthy United States adult population (NCT03827395) was sponsored by the National Institute of Allergy and Infectious Diseases (NIAID) in Atlanta, Georgia, with 25 participants [43]. They included male and non-female patients 18–45 years of age, who received doses at 0–1–6 months. Primary objectives were to assess the efficacy after each dose and any increase in HEV immunoglobulin G (IgG) ≥ 4-fold [44]. The results show that 13 out of the 20 participants had a 4-fold or greater rise in serum HEV IgG concentration after 15 days from the first dose. After the second dose on day 29, 100 percent of those who received the treatment showed a 4-fold or greater HEV IgG concentration. This widespread efficacy remained constant after the third dose at day 180 and until the end of the study on day 360. On the other hand, none of those who received the placebo exhibited any change in HEV IgG concentration throughout this time. Secondary outcomes included HEV immunoglobulin M conversion, IgG seroconversion, and IgG concentration changes [43]. The seroconversion of HEV IgM did not change significantly, as it occurred in three of the twenty participants who received the vaccine. None of these participants exhibited HEV IgM seroconversion after the third dose, which remained true for the rest of the study. However, 12 out of 20 from the treatment group had HEV IgG seroconversion 15 days after the first dose, and 100 percent of the treated participants after the second dose, which remained true for the rest of the study. The geometric mean shows that the treated participants’ HEV IgG concentration is typically highest 2–3 weeks after the administration of each dose. Furthermore, the HEV IgG concentration almost doubles after each subsequent dose.

The Phase 2 field efficacy trial of a candidate hepatitis E vaccine (Sar 56 kDa) (NCT00287469), sponsored by the U.S. Army Medical Research and Development Command and collaborators GlaxoSmithKline and NIAID in Kathmandu, Nepal, has 2000 participants [45]. Their primary outcome was to evaluate the safety and efficacy of the vaccine [44]. The study provided a similar dosage to the Phase 1 study, with dose 1 given at month 0, dose 2 at month 1, and dose 3 at month 6. Furthermore, the treatment group comprised half of the study population who received the vaccine, 20 mcg of recombinant HEV antigen, while the other half received the placebo. During the follow-up period from dose 2 to dose 3, 100 out of the 1000 participants who received the vaccine exhibited immunological markers (anti-HEV), had ALT greater than 2.5 times the upper normal limit, and displayed HEV symptoms for at least 3 days. In addition, this number increased to a total of 300 individuals 14 days after dose 3 of the vaccine. However, the study identified one case of HEV in the treatment group before the administration of dose 1. Furthermore, 18 cases of HEV infection were found in the treatment group during the time period of first dose administration to 14 days after the third dose. Of these 18 cases, 9 cases were definite Hep E disease, while the other 9 cases were probable or not confirmed. Within 31 days post-vaccination, 143 participants who received the vaccine had serious adverse events, accounting for approximately 14.3% of the treatment group. On the contrary, 200 individuals from the placebo group had serious adverse events, making up 20% of the placebo arm. Some of the biggest risk factors for both groups include infections and infestations such as HEV, leptospirosis, and typhoid fever. Notably, 81 individuals from the placebo group contracted HEV, while 10 participants from the treatment group had HEV infection. Furthermore, there were no deaths in the placebo group, whereas the treatment group had 6 deaths. Of these six deaths, one was from cholangiocarcinoma, four were from combat, and one was from an undetermined cause 130 days after the second dose. The data and safety board determined that the vaccine did not contribute to any of these deaths [45]. In addition, 44.5% of the study population experienced non-serious adverse events including diarrhea, abdominal pain, pyrexia, nasopharyngitis, and headache. However, there was no significant difference in the number of people who experienced non-serious adverse events between the placebo and treatment group.

### 5.2. Current Active Studies, Not Recruiting

A study on the immunogenicity and safety with co-administration with recombinant human papillomavirus 16/18 bivalent vaccine and HEV (Cecolin^®^ and Hecolin^®^) is being sponsored by Xiamen Innovax Biotech Co. in Hangzhou, Zhejiang, China, with an estimated enrollment of 480 participants [46]. The efficacy of co-administration of the two vaccines is the primary outcome [46].

### 5.3. Current Studies Recruiting

Long-term Effectiveness of a Recombinant Hepatitis E Vaccine: a Test-negative Design Study (NCT05976594) is currently recruiting out of Dongtai, Jiangsu, China, with an estimated enrollment of 2900 participants [47]. The primary outcome is to evaluate residual clinical serum from patients born between 1941 and 1991, with ALT levels >2.5 times the upper limit of normal in this region. They will then assess hepatitis E infection, as well as review HEV vaccination history to evaluate the long-term effectiveness of the HEV 239 vaccine [47].

### 5.4. Current Studies Not Yet Recruiting

The Phase 2 immunogenicity trial of Hecolin^®^ in healthy pregnant women with gestational age 14–34 weeks and non-pregnant women 16 to 45 years old (NCT05808166), sponsored by the International Vaccine Institute in Karachi, Sindh, Pakistan, has an estimated enrollment of 2358 participants [48]. The primary goal of the study is the safety and efficacy of the HEV vaccine, with the secondary endpoints of immune response through passive immunization from transplacental transfer [48]. Patients will follow the 0–1–6-month immunization schedule, but doses 1 and 2 will be peripartum and dose 3 will be postpartum [48].

## 6. Use in Special Populations

### 6.1. Pregnant Women

While the use of HEV vaccination in non-pregnant women of childbearing age has been well-studied and has provided reassuring evidence on the safety and efficacy of HEV vaccination, evidence for use in pregnant women has been scarcer. Considering the high rates of mortality of pregnant women who become infected with virulent genotypes of HEV [49], the applicability of HEV vaccination in this population is of great importance. Although universal vaccination of all women before childbearing age or pregnancy in endemic regions could be an ideal solution, practical application is likely extremely difficult considering that most regions where HEV genotype 1 is endemic are also low-income countries where healthcare resources must be triaged.

The major concern regarding vaccination during pregnancy is harm to the developing fetus due to a pathogenic live virus. Thus, live vaccines are never used during pregnancy [50]. The only HEV vaccine approved for general use, HEV 239, is a recombinant vaccine and thus has no risk of causing an HEV infection in the mother. However, real-world evidence of the vaccine’s safety in pregnant women is scarce, as clinical trials involving pregnant women, in general, are considered to be ethically and medically complicated. In Phase 3 clinical trials of HEV 239 in China from 2007 to 2009 involving over 112,000 participants, there were 37 women in the experimental group and 31 women in the placebo group who were unknowingly pregnant and received the HEV 239 vaccine and placebo vaccine, respectively. Some of the women in both groups decided to undergo elective abortion, 19 in the experimental group and 14 in the placebo group. Among those women in both groups who decided not to undergo elective abortion, all delivered live births. There were no differences in the weight, body length, and gestational ages between the infants born from women in the experimental and placebo groups [51]. More recently in 2020, a Phase 4 clinical trial in Bangladesh, which is estimated to involve 20,000 non-pregnant women of childbearing age, is investigating the effectiveness, safety, and immunogenicity of the HEV 239 vaccine and is expected to provide more data on the vaccine’s safety in women who are incidentally pregnant during vaccine administration [52].

### 6.2. Chronic Viral Hepatitis and Chronic Liver Disease Patients

Patients who have chronic liver disease, including chronic viral hepatitis, are at risk of developing a severe condition called acute-on-chronic liver failure. One of the acute insults that could trigger this condition is HEV infection [53]. This is of particular concern for HEV vaccines that use a live virus. Currently, the only approved HEV vaccine for human use, HEV 239, is a recombinant vaccine and therefore has no theoretical risk of triggering acute-on-chronic liver failure in patients with chronic liver disease. Additionally, the latest American and European practice guidelines for acute-on-chronic liver failure make no mention of any vaccines being a risk or trigger for developing that condition [53,54]. In fact, the American practice guidelines by Bajaj et al. even recommend that patients with chronic liver disease be vaccinated with the hepatitis A and hepatitis B vaccines [54]. In a study by Wu et al. investigating the efficacy of HEV 239 in participants with hepatitis B surface antigen (HBsAg) positivity, the authors found that the safety and immunogenicity of HEV 239 for participants with HBsAg positivity to be very similar to that for the general population [55]. While the use of HEV 239 in people with severe liver fibrosis or cirrhosis is expected to be safe, direct evidence for this population is lacking, and further study is worthwhile if acute hepatitis E infection may put this population at risk for liver decompensation.

### 6.3. Immunosuppressed and Immunocompromised Patients

In patients who are immunosuppressed, particularly in solid-organ transplant recipients, a unique phenomenon occurs where HEV infection, which is typically acute, can become chronic. This phenomenon has been reported in developed countries where HEV genotype 3 is endemic. HEV genotype 3 infection is almost always asymptomatic or mild and self-limited in the general population; however, in solid-organ transplant recipients, the infection can become chronic and result in chronic hepatitis, cirrhosis, and even possible rejection of the transplanted organ [56,57,58,59,60]. In immunocompromised patients, such as patients with HIV, particularly those with a very low CD4+ count (<200 cells/mm^3^), chronic HEV infection can also occur and result in similar complications [61,62]. Thus far, there have been no data on the safety and efficacy of HEV vaccines in immunocompromised or immunosuppressed populations. Currently, HEV vaccines remain a niche product, and scientific and financial incentives and motivations to further develop and test the product in special populations are limited. As all current and in-development vaccines for HEV are non-live vaccines, there are no theoretical contraindications to using HEV vaccines in populations that are immunocompromised or immunosuppressed. Similar to other non-live vaccines given to immunocompromised or immunosuppressed individuals, more frequent boosters for HEV vaccines are likely needed to maintain effective immunity.

As such, there is a need for a further study of HEV vaccine use in these populations. However, there is lack of data on HEV vaccines in solid-organ transplant recipients and in immunocompromised patients such as those with HIV; the long-term efficacy, duration of protection, and the timing of the booster injection need to be investigated [63].

## 7. Conclusions

In conclusion, the ongoing efforts to develop an effective vaccine against hepatitis E emphasize the critical need to address the complexities of the disease, particularly in vulnerable populations such as pregnant individuals and those with compromised immune systems. The Phase 2 immunogenicity trial of Hecolin^®^ in Pakistan represents a significant step forward in evaluating the safety and efficacy of the vaccine in pregnant women and exploring passive immunization through transplacental transfer. However, further research is warranted to assess the vaccine’s safety and efficacy in populations such as those with severe liver fibrosis or cirrhosis, solid-organ transplant recipients, and immunocompromised patients. Long-term studies are necessary to determine the duration of protection and optimal timing for booster injections. By advancing our understanding and addressing the specific needs of these populations, we can better mitigate the impact of hepatitis E and improve outcomes for those at greatest risk.

## Figures and Tables

**Table 1:** Current and ongoing clinical trials.

Phase of Clinical Trial	Author(s)	Study Period	HEV Vaccine Name	Vaccine Type	Study Sample Size	Location	Key Findings
Completed without results	Zhang [34]	2007–2017	Hecolin	Recombinant (*E. coli*)	112,604	Jiangsu, China	N/A
	Zhang [35]	2014–2015	Hecolin	Recombinant (*E. coli*)	601	Fujian, China	N/A
	Chen [36]	2017–2018	Hecolin	Recombinant (*E. coli*)	125	Zhejiang, China	N/A
	Jiangsu Province CDC [37]	2015–2016	Hecolin	Recombinant (*E. coli*)	60	Jiangsu, China	N/A
	Chen [38]	2015–2016	Hecolin	Recombinant (*E. coli*)	600	Zhejiang, China	N/A
	Hu [39]	2018–2019	Hecolin	Recombinant (*E. coli*)	360	Jiangsu, China	N/A
	Zhang [40]	2015–2016	Hecolin	Recombinant (*E. coli*)	602	Beijing, China	N/A
	Dudman [41]	2017–2019	Hecolin	Recombinant (*E. coli*)	19,460	Dhaka, Bangladesh	N/A
	Wu [42]	2012–2019	Hecolin	Recombinant (*E. coli*)	7372	Beijing, China	N/A
Completed with results	Xu [43]	2016–2017	Hecolin	Recombinant (*E. coli*)	475	Shandong, China	A/Es similar between control and experimental group
	NIAID [44]	2019–2020	Hecolin	Recombinant (*E. coli*)	25	Georgia, United States	Efficacy lasted 360 days after 3 doses of vaccine
	Shrestha, Scott [45]	2001–2005	Sar 56 kDa	Recombinant (*E. coli*)	2000	Kathmandu, Nepal	No statistically or clinically significant differences between groups for non-serious A/Es
Current active studies, not recruiting	Jiang [46]	2021–2023	Hecolin	Recombinant (*E. coli*)	480	Zhejiang, China	N/A
Current studies recruiting	Jun [47]	2023–Present	Hecolin	Recombinant (*E. coli*)	2900 (Estimated)	Jaingsu, China	N/A
Current studies not yet recruiting	International Vaccine Institute [48]	2024–Present	Hecolin	Recombinant (*E. coli*)	2358 (Estimated)	Karachi, Sindh, Pakistan	N/A

Legend: CDC, Centers for Disease Control and Prevention; A/Es, Adverse effects; NIAID, National Institute of Allergy and Infectious Diseases; N/A, not applicable.
